# Living with dementia in a care home: facilitating participation in activities

**DOI:** 10.3389/fspor.2025.1563025

**Published:** 2025-08-08

**Authors:** Katarina Galof

**Affiliations:** Faculty of Health Sciences, Occupational Therapy Department, University of Ljubljana, Ljubljana, Slovenia

**Keywords:** occupational therapy, dementia, quality of life, activities, care home

## Abstract

**Introduction:**

In Slovenia, about 5% of people over 65 live in care homes. Although there is no accurate data on the prevalence and incidence of dementia, the number of people with dementia is increasing, mainly due to ageing. As life expectancy increases, so does the prevalence of dementia, which poses major challenges for ageing societies, including Slovenia. It is estimated that more than 40,000 people currently live with dementia in Slovenia. Occupational therapists are integral members of care teams in care homes. Their job is to provide residents with meaningful activities and improve quality of life.

**Aim:**

The aim of this study was to investigate the frequency of different methods and techniques used by occupational therapists in the care of people with dementia in care homes.

**Methods:**

An online survey was conducted, targeting occupational therapists working in residential care facilities. The questionnaire, developed based on professional experience and knowledge of occupational therapy in such facilities, used a 5-point Likert scale (never, occasionally, often, very often, always) to assess the frequency of strategies and techniques used in dementia care.

**Results:**

The results showed differences in the use of therapeutic strategies. In Slovenia, occupational therapists often focus on training and enabling people with dementia to be involved in activities of daily living, while in other countries more emphasis is placed on training carers and health professionals and including people with dementia in leisure activities, including sports.

**Discussion:**

Occupational therapists play a crucial role in improving the quality of life of people with dementia. Training in this area may be inadequate, which could explain the lower integration of sports and similar activities into therapy. Another factor could be the stage of dementia at which such activities are no longer suitable without modifications. A deeper investigation into reasons for the limited use of sports in dementia care by occupational therapists is warranted. It is crucial to recognize that each person with dementia experiences physical activity differently and faces unique challenges. Occupational therapy can greatly enhance their quality of life.

## 1 Introduction

Ageing is an inevitable and lifelong process that is unique to each person. It reflects the complex interplay of biological, psychological and environmental factors. Each person experiences the ageing process differently, influenced by genetics, lifestyle and living conditions. Factors that influence the ageing process include climate, urban living, unhealthy habits, poor diet, occupational stress and lack of physical activity. In contrast to chronological ageing, these aspects contribute to biological and experiential ageing, which can be positively influenced by a healthy, active lifestyle, maintaining emotional stability and fostering good relationships throughout the lifespan (1).

Life expectancy is increasing worldwide, and the population is ageing at an unprecedented rate. According to the World Health Organization, the number of people aged 60 and over will increase from 1 billion in 2020 to 1.4 billion in 2030. By 2050, this number is expected to double and reach 2.1 billion, with the number of people aged 80 and over tripling to 426 million (2). This demographic change will have a profound impact on healthcare systems, social structures and economies worldwide.

These trends are particularly pronounced in Slovenia. With a population of around 2 million inhabitants, Slovenia is ageing faster than the European Union average. Currently, more than 21% of the population is aged 65 or over, and this proportion is expected to rise to almost a third by 2050. This demographic change is mainly due to increasing life expectancy and falling birth rates among younger generations (3).

The Slovenian government has proactively addressed these challenges with policy measures such as the Strategy for Active Ageing adopted in 2017. This strategy aims to create a society that is inclusive of all generations and emphasizes cooperation and solidarity between generations. The framework advocates technological advances, adjustments in the labor market and a redefined understanding of work-life balance to address demographic changes. It promotes a positive vision of longer life expectancy and emphasizes the importance of purposeful and active ageing.

Building on these foundations, Slovenia adopted its second national strategy for dealing with dementia in 2023 (4). This strategy, which extends to 2030, represents a comprehensive and integrated approach to dementia care. Its goals include ensuring accessible, high-quality care and improving the quality of life for people with dementia, their families and carers. The strategy comprises ten objectives, including: Promoting prevention programmes to reduce risk factors; improving early diagnosis and access to healthcare for neurocognitive disorders; providing multidisciplinary and coordinated care; using modern information and communication technologies to support dementia care; reducing stigma, raising awareness and promoting dementia-friendly communities; training professionals to deal with dementia; introducing a national data collection on dementia; promoting dementia-related research; creating a national dementia centre; and ensuring appropriate care during epidemics and emergencies.

The implementation of the strategy is monitored by the Ministry of Health, with action plans covering 2–5 years. The first plan (2023–2024) focuses on the development of expert bases and the promotion of ongoing initiatives (4).

The prevalence of dementia in Slovenia emphasizes the urgency of such initiatives. In 2018, around 1.65% of the population (34137 people) lived with dementia, a figure that is expected to rise to 3.65% (65892 people) by 2050. This trend mirrors the general development in Europe, where the number of people with dementia is expected to almost double by the middle of the century (5).

A key driver of this increase is the growing population aged 65 and over, particularly those aged 85 and over, whose numbers are expected to more than double in Slovenia between 2018 and 2050. This demographic change poses challenges for the Slovenian health and social care system that require innovative and sustainable solutions.

Currently, the Slovenian long-term care system focuses on home-based services, including social home care and community healthcare. These services enable older adults to maintain their independence and remain in their homes, reducing the need for institutional care. Home care (Ageing in Place), which is managed by municipalities, provides support with activities of daily living, household tasks and socializing. In 2021, these services reached almost 13000 users in 211 municipalities, served by 76 organizations and over 1100 direct service providers (6).

Occupational therapists play a central role in this context. Despite their important contribution, Slovenia faces the challenge of meeting the demand for occupational therapy services. There are 630 practicing occupational therapists in the country, which equates to 30 per 100000 inhabitants. This number is lower than in other European countries such as Switzerland (38 per 100000), Austria (53 per 100000), Finland (70 per 100000) and Germany (78 per 100000) (7).

The World Federation of Occupational Therapists (8) defines occupational therapy as a person-centred healthcare profession that focuses on enabling people to participate in meaningful activities. Occupational therapists assist people with everyday tasks such as bathing, dressing and eating, often using assistive devices to increase independence. They also provide training for carers, develop structured routines and support cognitive function, memory and executive skills.

In dementia care, occupational therapists are in a unique position to address the complex needs of those affected and their families. Their expertise includes developing tailored strategies to manage the behavioral and psychological symptoms of dementia, facilitating meaningful participation in activities to improve quality of life, preventing falls and assessing safety in the home, training caregivers in effective communication and support techniques, promoting cognitive stimulation, and maintaining functional abilities (9).

Occupational therapists play a central role in facilitating participation in meaningful daily and leisure activities, which helps people with dementia maintain a sense of identity, autonomy, and engagement. This includes adapting tasks to match the person's current abilities, modifying the environment to reduce barriers, and using motivational strategies to enhance involvement. Participation is not just about doing tasks—it's about being part of life in a way that has meaning for the person (10).

Additionally, occupational therapists contribute to the promotion of safe and regular physical activity, which has been shown to improve mobility, falls efficacy, and quality of life in people with dementia (11, 12). They may co-design individualized home-based or group-based exercise programs, work with care staff and families to improve adherence, and support digital solutions that enable continued participation, even during periods of social isolation (13).

Despite these contributions, outdated staffing levels in Slovenia pose a challenge. Currently, one occupational therapist cares for around 150 residents in care homes. This discrepancy highlights the need for systemic reforms to adapt resources to the growing demand for occupational therapy services.

Despite these valuable contributions, outdated staffing norms in Slovenia limit the practical implementation of occupational therapy in dementia care. Currently, one occupational therapist is responsible for approximately 150 residents in care homes, which significantly exceeds recommended caseloads. This discrepancy underscores the urgent need for systemic reforms and investment to align staffing levels with the growing demand for individualized and meaningful occupational therapy interventions in aged care settings.

Globally, the role of occupational therapists in dementia care is increasingly recognized (8). Numerous studies (14, 15) highlight that occupational therapy interventions can significantly improve outcomes for people with dementia—such as enhanced daily functioning, reduced caregiver burden, and improved mental well-being. For instance, personalized activity programs have been shown to reduce agitation and depressive symptoms while promoting a sense of identity and purpose (16, 17). In addition, environmental adaptations—such as reducing clutter or improving lighting—can help reduce confusion and enhance safety in the living space (18, 19).

Recent systematic reviews and meta-analyses continue to affirm the critical role of occupational therapy in dementia care. A 2018 narrative synthesis highlighted those interventions delivered by occupational therapists such as personalized activity engagement, environmental modifications, and sensory stimulation, are effective in reducing behavioral and psychological symptoms of dementia, enhancing daily functioning, and improving quality of life in long-term care residents (20). Similarly, a 2021 meta-analysis evaluating the Tailored Activity Program (TAP) confirmed its effectiveness in reducing neuropsychiatric symptoms and caregiver burden, while improving functional abilities among people living with dementia (21).

Beyond occupational therapy, a wide range of non-pharmacological interventions has demonstrated effectiveness, including music therapy, reminiscence therapy, cognitive stimulation therapy (CST), physical exercise programs, and the use of digital technologies such as socially assistive robots and ICT-based applications (22). In the Slovenian context, Kejžar (23) emphasizes the importance of using stimulating activities, such as daily tasks, movement, cognitive training, and creative expression, as a central tool in social work with people with dementia. These interventions contribute to maintaining cognitive and emotional function, delaying deterioration, and improving well-being.

Within this interdisciplinary framework, occupational therapists hold a distinct and integrative role by tailoring such interventions to each individual's physical, cognitive, and psychosocial profile. Their practice promotes autonomy, safety, and meaningful engagement in everyday life. This aligns directly with the priorities set by Slovenia's National Dementia Strategy, which advocates for person-centered, coordinated, and community-based care approaches in response to the growing needs of the ageing population.

### 1.1 Aim

This study examines the methods and techniques used by occupational therapists in dementia care in care homes (nursing homes, retirement homes) in Slovenia. By examining the frequency and effectiveness of these interventions, the study aims to provide insights into best practise and identify areas for improvement. The results will contribute to the further development of the Slovenian long-term care system and support the goal of providing high-quality, accessible and sustainable care for people with dementia.

## 2 Methods and materials

We used quantitative research methods. The data for the study were collected using an online questionnaire developed based on knowledge about the work of occupational therapists in care homes for older adults in Slovenia and a review of the professional and scientific literature.

### 2.1 Participants

The online questionnaire was created using the 1KA survey tool and distributed via email to 127 addresses obtained from the publicly available Register of Social Institutions, managed by the Association of Social Institutions of Slovenia (24). These 127 addresses corresponded to all officially registered care homes in the country and represented the full institutional landscape relevant for long-term dementia care. The aim was to reach occupational therapists working specifically in institutional elderly care, not across all fields of occupational therapy in Slovenia. While there are approximately 630 practicing occupational therapists in Slovenia, this figure includes therapists working in various settings, such as hospitals, schools, rehabilitation centers, and community services. Therefore, the 127 institutions targeted were not a sample of individuals, but rather a complete enumeration of the relevant care homes (i.e., a full population approach within that context).

Care home managers were contacted in advance to request permission to recruit their occupational therapists and were provided with a survey link to forward to relevant staff. However, no feedback was received regarding how many managers forwarded the questionnaire to their occupational therapists. Participation was entirely anonymous and voluntary, and managers retained the discretion to decide whether to encourage their employees to participate.

A total of 83 completed responses were received. Since we could not verify how many therapists received the invitation, it was not possible to calculate a precise response rate or conduct a formal statistical power analysis. Nevertheless, the responses covered a broad geographic and institutional distribution, which suggests a degree of representativeness for occupational therapists working in Slovenian long-term care settings. However, results should still be interpreted with caution due to the unknown denominator and potential response bias.

Two inclusion criteria were applied: participants had to be (1) qualified occupational therapists and (2) employed in a care home setting at the time of data collection.

#### 2.1.1 Ethical considerations

The study adhered to general ethical research principles, including informed consent, voluntary participation, and data confidentiality. Completion of the questionnaire was considered implied consent. As no personal or sensitive data were collected and participation was anonymous, the ethical risk was minimal. The recruitment process involved contacting care home managers, who were given full information about the nature and purpose of the study. These managers had the opportunity to independently assess whether participation aligned with the ethical standards and internal policies of their institutions. Based on this judgement, they could decide whether to forward the survey link to their occupational therapy staff. This approach ensured institutional autonomy and respected managerial responsibility in the recruitment process (25, 26).

### 2.2 Data collection

The questionnaire collected demographic information about the participants. To preserve anonymity, respondents were not asked to provide the name or type of their facility, as Slovenia distinguishes between public, private, and specialised care homes for older adults. The inclusion criterion followed the national definition of institutional care for older adults in Slovenia, which “serves to alleviate the personal hardships and difficulties of persons over 65 years of age and other persons who cannot live at home due to illness, old age or other reasons. Care homes replace or supplement domestic and family functions by providing accommodation, organised meals, protection and healthcare” (27).

Regarding occupational therapy, the questionnaire included items on the number of years participants have worked in the profession, their length of employment in their current institution, the average amount of time they spend with people with dementia, and whether they had received additional training for working with this population.

The survey was open during July and August 2024. In the final week of August, a reminder email was sent to encourage participation. The collected data were analysed using IBM SPSS Statistics 25. Descriptive statistics were calculated, and skewness and kurtosis values were used to test the normality of distributions. Chi-square tests and independent samples t-tests were used to explore associations and differences between variables, with the significance level set at *p* < 0.05.

Although a formal pilot study was not conducted as part of this research, the questionnaire was previously pilot tested as part of a bachelor's thesis, which helped refine the structure and clarity of the items. This prior use provides a degree of content validity. Nevertheless, for future research, it is recommended that formal pilot testing be conducted, including reliability analysis and psychometric evaluation, in line with best practices for scale development and validation (28, 29).

### 2.3 Data analyzed

A total of 83 occupational therapists participated in the study, of whom 6% (*N* = 5) were male. Sixty-five (78.3%) of the participants had been working in occupational therapy for more than five years. Fifty-three (63.9%) had been providing occupational therapy interventions with dementia patients for more than five years.

Most occupational therapists (*N* = 74, 89.2%) reported working less than 20 h per week with people with dementia, while a smaller proportion (*N* = 9, 10.8%) spent 20 h or more per week, with a standard working time of 40 h per week. In terms of weekly workload, occupational therapists reported working with up to 20 different people (*N* = 40, 48.2%), of whom 69.9% (*N* = 58) were categorized as having moderate dementia (Table 1).

**Table 1:** Demographic characteristics of the participants (*N* = 83) and other variables.

Variable	Category	*N* (%)
Sex	Male	5 (6.0)
	Female	78 (94.0)
Years of occupational therapy professional practice	Less than 1 year	3 (3.6)
	From 1 to 5 years	15 (18.1)
	More than 5 years	65 (78.3)
Years of practicing occupational therapy for persons with dementia	Less than 1 year	4 (4.8)
	From 1 to 5 years	26 (31.3)
	More than 5 years	53 (63.9)
Completed additional training for working with dementia	Yes	58 (69.9)
	No	25 (30.1)
Occupational therapy practice includes people in different dementia stages	Early stage of dementia	10 (12.0)
	Middle stage of dementia	58 (69.9)
	Later stage of dementia	15 (18.1)
Weekly working time dedicated to people with dementia	Less than 20 h	74 (89.2)
	20 h or more	9 (10.8)
Weekly working time for education of team members/relatives	Less than 5 h	72 (86.7)
	5 h or more	11 (13.3)
Number of people with dementia engaged in weekly occupational therapy practice	Less than 5	8 (9.6)
	From 6 to 20	35 (42.2)
	More than 21	40 (48.2)

## 3 Results

### 3.1 Evaluation—use of structured assessment instruments

Occupational therapists use some measurements in an evaluation process for people with dementia as instruments to document and assess the problems and evaluate the effects of the occupational therapy intervention (Table 2).

**Table 2:** Use of assessment tools by participants (*N* = 83).

Variable	*N* (%)	*N* (%)	*N* (%)	*N* (%)	*N* (%)
Assessment instrument	Rarely	Occasionally	Often	Always	Never
MMSE	4 (4.8)	13 (15.7)	12 (14.5)	34 (41)	20 (24.1)
CDT	8 (9.6)	18 (21.7)	15 (18.1)	23 (27.7)	19 (22.9)
FIM	24 (28.9)	23 (27.7)	11 (13.3)	15 (18.1)	10 (12.0)
BI	24 (28.9)	29 (34.9)	15 (18.1)	8 (9.6)	7 (8.4)
MBI	28 (33.7)	29 (34.9)	10 (12.0)	7 (8.4)	9 (10.8)
AMPS	33 (39.7)	25 (30.1)	9 (10.8)	8 (9.6)	8 (9.6)
AA	70 (84.3)	13 (15.7)	0 (0.0)	0 (0.0)	0 (0.0)
OSA	44 (53.0)	16 (19.3)	15 (18.1)	5 (6.0)	3 (3.6)
IC	44 (53.0)	18 (21.7)	9 (10.8)	8 (9.6)	4 (4.8)
OQ	41 (49.4)	20 (24.1)	4 (4.8)	5 (6.0)	13 (15.7)
IT	23 (27.7)	9 (10.8)	25 (30.1)	10 (12.0)	16 (19.3)

MMSE, mini-mental state examination; CDT, clock drawing test; FIM, functional independence measure; BI, the barthel index; MBI, modified barthel index; AMPS, assessment of motor and process skills; AA, activity analysis; OSA, the occupational self-assessment; IC, interest checklist; OQ, occupational questionnaire; IT, facility's internal tests.

Evaluation, which is an integral part of the occupational therapy service delivery process, is conducted by occupational therapists both at the beginning of the occupational therapy service delivery process for people with dementia and at the end when determining whether or not goals have been achieved (10).

In the field of treating people with dementia, occupational therapists most commonly use three assessment tools: Mini Mental State Examination (MMSE) (*N* = 54, 64.1%), Clock drawing test (CDT) (*N* = 42, 50.6%) and Functional Independence Measure (FIM) (*N* = 25, 20.1%). All other assessment instruments listed in Table 2 were rarely or sometimes used. MMSE and CDT are assessment tools intended for the assessment of cognitive impairment in people with dementia.

When using the FIM assessment tool, occupational therapists observe and evaluate the level of performance of people with dementia in performing activities of daily living (ADL) (self-care, mobility, locomotion, communication). Twenty-six (31.3%) occupational therapists also use the facility's internal tests (IT) and 18 (21.7%) use the Occupational Questionnaire (OQ).

We also analyzed the correlation between the variables relating to the use of assessment instruments (1 = never/rarely/occasionally, 2 = often, 3 = always) and the acquisition of additional professional knowledge for working with people with dementia (1 = yes, 2 = no) using the *χ*^2^ test. The results show no significant correlation between the variable of additional knowledge acquired and the use of the KPSS assessment tool (*χ*² = 0.169, *p* = 0.919). However, a significant correlation was found between the variable of additional knowledge acquired and the use of the CDT assessment tool (*χ*² = 13.652, *p* = 0.001) at the 5% level of specificity.

### 3.2 Types of strategies and interventions used by occupational therapists

The intervention process includes services provided by occupational therapists in collaboration with people with dementia to promote participation in meaningful activities (10). Occupational therapists most frequently use interventions such as cognitive stimulation training (*N* = 71; 85.6%), ADL training (*N* = 66; 79.5%), implementation of social integration strategies (*N* = 58; 69.9%) and creative approaches to enable participation in craft activities (*N* = 49; 59.0%) (Table 3).

**Table 3:** Strategies and interventions used by participants (*N* = 83).

Variable	*N* (%)	*N* (%)	*N* (%)	*N* (%)	*N* (%)
OT strategies/interventions	Never	Rarely	Occasionally	Often	Always
Training of ADL	0 (0.0)	4 (4.8)	13 (15.7)	24 (28.9)	42 (50.6)
Training of IADL	42 (50.6)	32 (38.6)	8 (9.6)	1 (1.2)	0 (0.0)
Prevent cognitive impairment (Brain training)	0 (0.0)	0 (0.0)	12 (14.5)	34 (41.0)	37 (44.6)
Prevent social isolation (Social inclusion)	0 (0.0)	5 (6.0)	20 (24.1)	36 (43.4)	22 (26.5)
Establish creative activities	0 (0.0)	6 (7.2)	28 (33.7)	40 (48.2)	9 (10.8)
To restore/remediate physical functioning (Recreational activities intervention)	0 (0.0)	12 (14.5)	45 (54.2)	26 (31.1)	0 (0.0)
Training relaxation techniques	0 (0.0)	44 (53.0)	39 (47.0)	0 (0.0)	0 (0.0)
Spatial orientation strategies	0 (0.0)	31 (37.3)	52 (62.7)	0 (0.0)	0 (0.0)
Environmental modification	71 (85.5)	11 (13.3)	1 (1.2)	0 (0.0)	0 (0.0)
Assistive technology	66 (79.5)	17 (20.5)	0 (0.0)	0 (0.0)	0 (0.0)
Education and consultation	22 (26.5)	61 (73.5)	0 (0.0)	0 (0.0)	0 (0.0)
Self-regulation (sensory stimulation)	4 (4.8)	38 (45.8)	40 (48.2)	1 (1.2)	0 (0.0)

ADL, activities of daily living; IADL, instrumental activities of daily living.

### 3.3 Participation of people with dementia in individual or group activities

**Figure 1:**
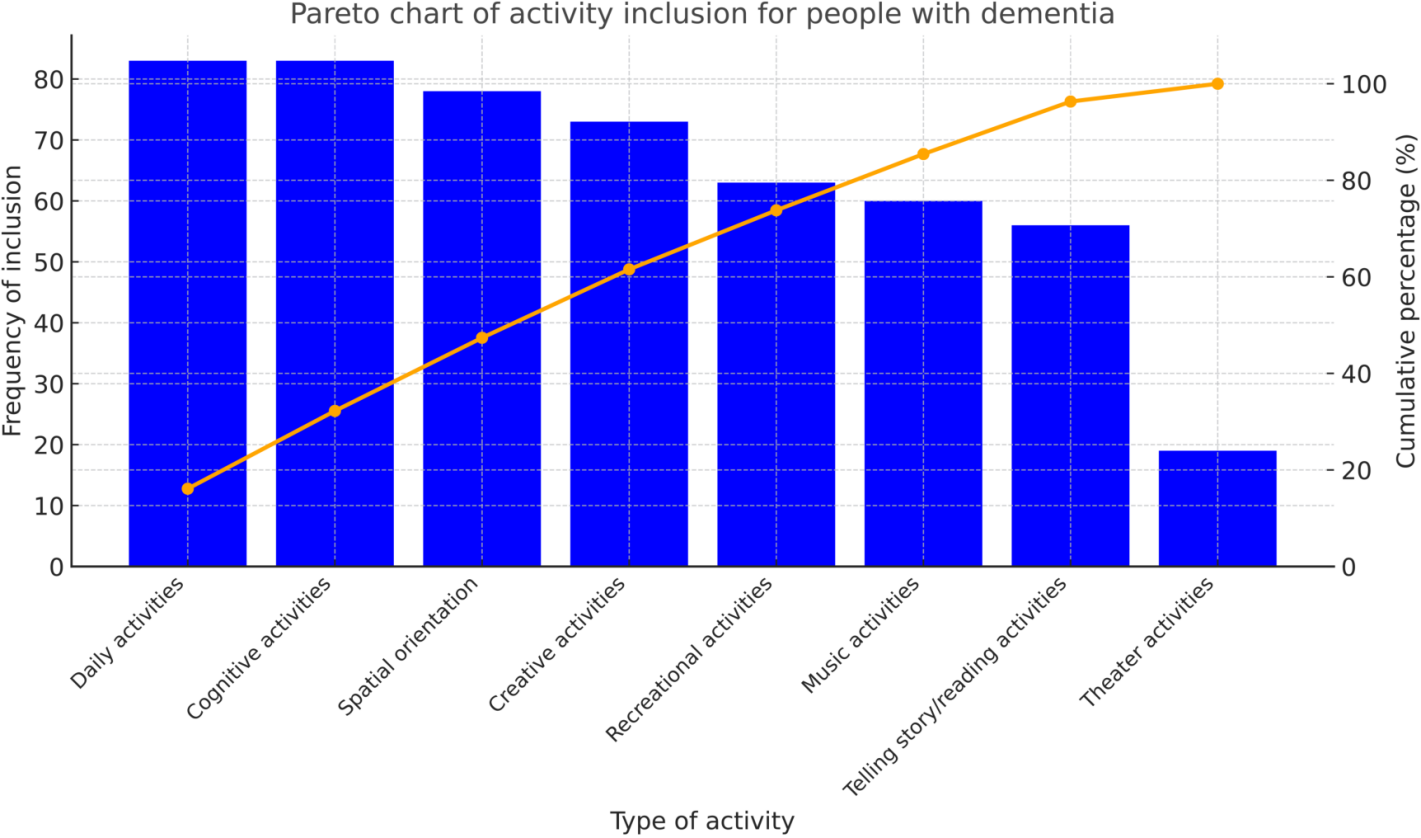
Pareto chart of people with dementia's activity inclusion. Source: author's own work.

Occupational therapists use occupations as a central aspect of their practice domain (10). Figure 1 illustrates a wide range of occupations categorized under activities such as rest and sleep, education, work, play, leisure and social participation.

The Pareto chart, which is sorted in descending order from highest to lowest frequency, visually represents the activities in which people with dementia participate. The height of the bars reflects the frequency or impact of each activity and indicates areas where occupational therapy interventions could be improved to enhance participation.

Occupational therapists most frequently involve people with dementia in activities of daily living and cognitive activities (*N* = 80). Another critical intervention from an occupational therapy perspective is the promotion of orientation in the institutional environment (*N* = 78). In Slovenian care homes, craft activities have always had a cultural significance for older adults. However, as the results show, arts and crafts are currently less emphasized by occupational therapists (*N* = 73). Conversely, people with dementia have the fewest opportunities to participate in theatre activities (*N* = 19). Leisure activities (*N* = 63) and musical activities (*N* = 60) are frequently used to facilitate individual or group participation.

A statistically significant difference was found in the use of the Functional Independence Measure (FIM) evaluation tool based on the stage of dementia [*p* = 0.001 < 0.05, t(df = 81) = 7.191]. On average, the FIM is more frequently used for individuals in the early and middle stages of dementia (M = 2.84) compared to those in the late stage (M = 1.33).

Additionally, the average involvement of individuals with dementia in ADL differs significantly between the early and middle stages (M = 4.31) and the late stage (M = 4.00) [*p* = 0.001 < 0.05, t(df = 81) = 0.880].

No statistically significant differences were observed in the use of recreational activity interventions (*p* = 0.460) or relaxation training techniques (*p* = 0.125) as part of the occupational therapy process for individuals with dementia.

## 4 Discussion

This section critically examines the key findings of the study in relation to existing literature and international best practices in occupational therapy for people living with dementia. The aim is to contextualize the results within current evidence, identify strengths and limitations in Slovenian occupational therapy practice, and outline potential directions for improvement and further development.

### 4.1 The role of occupational therapists in Dementia Care

Occupational therapists play a vital role in supporting people with dementia to participate in daily activities that are meaningful and purposeful. These occupations serve not only as therapeutic tools but also as essential outcomes in dementia care, contributing to improved quality of life (10, 30). Since people with dementia are often functionally dependent and physically inactive (11), occupational therapy interventions are essential in promoting engagement, independence, and overall well-being.

In Slovenia, therapists employ both individual and group-based approaches, with group interventions being more common (77.1%), reflecting organizational realities within institutional settings. These interventions can take place in a variety of environments, at home, in the community, or in long-term care facilities, and may involve family members or caregivers as part of the therapeutic process (10).

According to the Occupational Therapy Practice Framework (OTPF-4), meaningful activities are categorized into domains such as ADLs, IADLs, health management, rest and sleep, education, work, play, leisure, and social participation. Of these, five domains are particularly relevant for older adults with dementia: ADLs, IADLs, rest and sleep, leisure, and social participation (10, 30).

However, as Fraker et al. (30) noted, occupational therapists working in home care settings often neglect certain OTPF-4 domains. The present study supports this observation, showing that rest and sleep (*N* = 2, 2.4%) and play (*N* = 0) are seldom included in therapeutic programs. In contrast, the most addressed areas include ADLs (81.9%), leisure (68.7%), social participation (59.0%), IADLs (55.4%), and work (41.0%). This pattern highlights a pragmatic orientation in occupational therapy, whereby interventions are focused on immediate functional needs and adapted to the abilities and priorities of prople with dementia.

### 4.2 Use of assessment tools

The most frequently used assessment tools among Slovenian occupational therapists are the Mini-Mental State Examination (MMSE; 65.1%) and the Clock Drawing Test (CDT; 50.6%), which align with international standards for cognitive screening (31–33). However, instruments that evaluate broader aspects of occupational participation, such as the Occupational Self-Assessment (OSA) and the Assessment of Motor and Process Skills (AMPS), remain significantly underutilized (34, 35). Similarly, the Functional Independence Measure (FIM), an internationally validated tool used to monitor changes in activities of daily living, is employed by only 30.1% of respondents, thereby limiting its potential for tracking intervention outcomes over time (36).

Although 69.9% of therapists reported receiving additional training in dementia care, the range of assessment tools in practice remains unexpectedly narrow (Table 2). This suggests a notable gap between continuing professional development and its application in everyday clinical settings. As Kristensen et al. (35) point out, many widely used tools prioritize functional status over participation or person-centred goals. Supporting this, the study found a statistically significant association between additional training and more frequent use of the CDT (*χ*^2^ = 13.652, *p* = 0.001), underscoring the impact of education on clinical decision-making.

The primary objective of the occupational therapy evaluation process is to determine what people with dementia want and need to do, and what they can do. This involves a comprehensive analysis of occupational performance as the basis for intervention planning (10). However, the findings of this study reveal a disconnect between available assessment tools and those commonly used in practice. For instance, in a German study, the MMSE was used as an inclusion criterion, targeting only individuals with mild to moderate dementia and thereby limiting the use of assessments focused on participation and engagement (33).

### 4.3 Intervention strategies and therapeutic approaches

The most applied interventions among Slovenian occupational therapists include cognitive stimulation (85.6%), training in activities of daily living (ADLs; 79.5%), and social participation (69.9%). These approaches are well-supported by international evidence and represent core components of effective dementia care (20, 21, 30). In contrast, interventions such as environmental modifications (1.2%) and the use of assistive technologies (0%) are rarely implemented, despite their established value in enhancing safety and ensuring person–environment fit (10, 37–39).

A particularly notable gap exists in caregiver education and training, which remains almost entirely absent from Slovenian occupational therapy practice. This is concerning given the emphasis placed on caregiver support in international guidelines (30, 40). The findings show that 86.7% of therapists spend less than five hours per week on caregiver or family education, suggesting systemic limitations or attitudinal barriers—such as ageism—may be hindering the adoption of more inclusive and holistic approaches (41).

Throughout the progression of dementia, occupational therapists aim not only to maintain functional abilities but also to support caregivers through education, communication skills training, and the facilitation of meaningful activities (30). In care home settings, individualized exercise programs are commonly used to promote physical activity, autonomy, and emotional stability. These programs often integrate elements such as movement, sensory stimulation, relaxation, and interpersonal interaction, tailored to the personal histories and preferences of individuals with dementia (40, 42).

When adapted to the individual's functional level, personalized exercise programs, whether in group or individual formats, can deliver significant benefits. These may include aerobic, strength, or flexibility components, and are often built around familiar activities such as gardening or household tasks, which increase engagement and promote therapeutic outcomes (12, 15, 43).

However, reducing sedentary behaviour in care homes remains a challenge. Co-developed interventions, created in collaboration with residents, caregivers, and staff, are essential to ensure feasibility and responsiveness to residents’ needs (44). For example, Frampton et al. (17) reported positive effects from chair-based yoga sessions in care homes. Additionally, various non-pharmacological approaches—including person-centred care, music therapy, communication training, meaningful activities, and sensory interventions, have proven effective in improving resident well-being and addressing common issues such as sleep disturbances (45–47).

Person-centred care, which prioritizes individual preferences, life stories, and tailored environments, has been shown to enhance trust and improve relationships between residents and staff (12). Occupational therapists play a central role in implementing these principles through daily routines, family involvement, and staff training. Key strategies include promoting regular physical activity, minimizing daytime inactivity, and establishing calming nighttime routines as part of a comprehensive, non-pharmacological care framework (46, 48).

### 4.4 Occupational participation and cultural context

Among people with dementia, participation is predominantly focused on activities of daily living (ADLs) and cognitively oriented tasks, while creative and expressive occupations—such as play, theatre, and the arts-are frequently neglected. This narrow focus stands in contrast to the principles of person-centred care, which emphasize the value of engaging individuals in meaningful, biographically rooted activities that support identity, emotional expression, and well-being (49, 50). Slovenian literature similarly underlines the therapeutic importance of culturally embedded occupations such as crafts, singing, and painting—activities that are strongly linked to individuals’ personal histories and cultural identity (34, 43, 51, 52).

Despite growing evidence advocating for the use of personalized and context-sensitive interventions, much empirical research fails to capture the complexity of real-life care environments. For instance, studies addressing spatial disorientation in individuals with Alzheimer's disease often lack ecological validity, limiting their practical applicability (53). Yet, it is precisely the contextual factors—such as familiar environments, meaningful relationships, and caregiver support—that play a crucial role in enhancing social participation and engagement in people with dementia (40).

Task-oriented interventions that are tailored to individuals’ abilities, preferences, and past experiences have been shown to produce superior functional and psychosocial outcomes (43). However, the implementation of such personalized activities is frequently challenged by systemic and individual barriers, including limited staffing, rigid institutional routines, and a lack of necessary resources (12). Addressing these obstacles is essential to enabling broader participation in diverse and meaningful occupations that align with the values of person-centred dementia care.

### 4.5 Physical activity and functional maintenance

Physical activity is widely recognized for its substantial benefits in maintaining mobility, cognitive function, and overall independence in people with dementia (12, 16, 43, 54). Defined as bodily movement initiated by skeletal muscles resulting in energy expenditure, physical activity has been shown to reduce functional decline, improve strength and endurance, and support cognitive functioning (16, 46).

In Slovenia, occupational therapists frequently implement task-oriented physical activities such as walking and gardening. However, relaxation techniques and sensorimotor approaches are less commonly employed, despite their demonstrated therapeutic value (42, 46). Tailored exercise programs—including yoga, swimming, strength training, and dancing have proven to be particularly effective when adjusted to individual capacities, limitations, and preferences (12).

Nevertheless, the practical implementation of these programs is often hindered by systemic factors such as institutional limitations, insufficient staffing, and time constraints (44). Furthermore, people with dementia are generally less physically active than their age- and gender-matched peers, which further increases the risk of functional decline (43, 44). In response, the Alzheimer's Society (55) has strongly advocated for the integration of physical activity into daily routines to support independence and maintain performance in daily living tasks (54).

Incorporating biographically meaningful and familiar activities—such as gardening, housework, or walking—not only enhances emotional engagement but also increases the overall therapeutic effectiveness of interventions (17, 33). Participation in ADL-related tasks is associated with improved outcomes; however, safety remains a critical concern, particularly regarding the proper use of mobility aids and fall prevention (43).

Mobility issues are prevalent among older adults with dementia, yet innovative solutions such as motor-cognitive training and the use of e-bikes have shown promise in supporting independence and facilitating social interaction (13, 56). These approaches underline the importance of creativity, personalization, and contextual awareness in designing effective physical activity programs within dementia care.

The delivery of holistic and person-centred dementia care in Slovenia is significantly constrained by systemic limitations. These include insufficient staffing—often with ratios as low as one occupational therapist per 150 residents—limited access to assistive technologies, and the near absence of structured caregiver education (13, 57). Overcoming these barriers requires institutional transformation and a broader commitment to occupation-centred, evidence-based practice frameworks (45, 47).

Person-centred interventions that involve family members, foster trust, and align with individual values and interests are essential to improving care outcomes. Recommended strategies include encouraging physical activity, implementing consistent daily routines, and creating calming environments to address behavioural symptoms and promote better sleep (46, 48).

Although Slovenian occupational therapists already apply many internationally validated methods in dementia care, notable gaps remain—particularly in the consistent use of standardized assessment tools, provision of caregiver education, and integration of environmental modifications. Nevertheless, Slovenia's strong cultural traditions offer a unique foundation for building more individualized and meaningful care. Therapeutic engagement through culturally embedded practices—such as art, crafts, music, and sport—enhances emotional expression, social connectedness, and cognitive stimulation (50–52).

For dementia care to be truly effective, interventions must be highly individualized and grounded in the person's biography. Activities like cycling, dancing, gardening, and housework not only reflect past experiences but also promote autonomy and engagement when delivered in small, socially supportive group settings (33, 43).

## 5 Conclusion

Aligning interventions with the interests, abilities, and life stories of people with dementia is essential for fostering participation and promoting an active, meaningful lifestyle. The integration of evidence-based practices, cultural responsiveness, and the systematic recording of successful approaches will support the ongoing advancement of occupational therapy in dementia care. Moving forward, strengthening collaboration between therapists, caregivers, and institutions will be critical to addressing systemic challenges and ensuring that dementia care in Slovenia becomes more inclusive, person-centred, and sustainable.

## Data Availability

The original contributions presented in the study are included in the article/Supplementary Material, further inquiries can be directed to the corresponding author.
