# Supplementary material for: Living with dementia in a care home: facilitating participation in activities
Source: Front Sports Act Living. 2025 Aug 8;7:1563025. doi: 10.3389/fspor.2025.1563025 (PMC12372530; doi:10.3389/fspor.2025.1563025)
Supplement: Supplementary file 1 [file Datasheet1.pdf]

Questionnaire: Occupational Therapy for People with Dementia

Introduction

Dear participant,

You are invited to take part in a survey about occupational therapy for people with dementia. The questionnaire is anonymous and all collected data will be used solely for research purposes. Please answer the questions sincerely and accurately.

Thank you for your time and cooperation.

Q1

How many years have you been working as an occupational therapist? (Single answer)

Q2

How many years have you been providing occupational therapy to people with dementia? (Single answer)

Q3

Have you completed any additional training or education for working with people with dementia? (Single answer: Yes/No)

Q4

On average, how many hours per week do you spend working directly with people with dementia? (Single answer)

Q5

On average, how many hours per week do you spend providing counselling to colleagues or family members of people with dementia? (Single answer)

Q6

Questionnaire: Occupational Therapy for People with Dementia

How many people with dementia do you work with on a weekly basis? (Single answer)

Q7

What is the typical stage of dementia of the people you most frequently work with? (Single answer: Mild, Moderate, Severe, Varies)

Q8

Indicate the frequency of using the following assessment tools: (Multiple answers in a table format)

Q9

In which areas of human occupation do people with dementia most often need intervention? (Multiple answers)

Q10

How often do you use the following techniques in therapy for people with dementia? (Multiple answers in a table format)

Q11

What form of therapy do you primarily use when working with people with dementia? (Single answer: Individual, Group, Combination)

Q12

Indicate the activities (group or individual) in which you include people with dementia: (Multiple answers)

Q13

What is your gender? (Single answer: Female, Male, Other, Prefer not to say)
